# Control of optics in random access analysers

**DOI:** 10.1155/S1463924688000367

**Published:** 1988

**Authors:** A. Truchaud

**Affiliations:** Biochimie Hôspital de Meaux Meaux F77104 France

## Abstract

The technology behind random access analysers involves flexible optical systems which can measure absorbances for one reaction at different scheduled times, and for several reactions performed simultaneously at different wavelengths. Optics control involves light sources (continuous and flash mode), indexing of monochromatic filters, injection-moulded plastic cuvettes, optical fibres, and polychromatic analysis.


Journal of Automatic Chemistry Vol. 10, No. 4 (October-December 1988), pp. 175-178

### Control of optics in random access analysers

**A. Truchaud**
*Biochimie, Hôpital de Meaux, F77104 Meaux, France*

*The technology behind random access analysers involves flexible optical systems which can measure absorbances for one reaction at different scheduled times, and for several reactions performed simultaneously at different wavelengths. Optics control involves light sources (continuous and flash mode), indexing of monochromatic filters, injection-moulded plastic cuvettes, optical fibres, and polychromatic analysis.*

### Introduction

Random access analysers can be defined as totally flexible, allowing multitests, organ panels, single tests and stats at any time and in any combination. An optical system is necessary to measure absorbances for one reaction at different scheduled times and for different reactions performed simultaneously at different wavelengths.

Only liquid phase reactions are discussed in this paper. If it was possible to consider only the reaction mixture, R, prepared by adding a sample to one or several reagents, the first controls would be the reagent('s) integrity control by a reagent blank and sample interferents by a sample blank at different wavelengths. The reaction could then be checked by absorbance measurements.

In reality the control of all the parts of the optical system has to be considered: these are the light source L, the monochromator, M, the optical cuvette, OC, the photocell, P, and the microprocessor, C.

### Control of light sources

It is necessary to distinguish between classic lamps, which emit light in a continuous mode, and flash lamps.

For the *continuous mode* (figure 1) the computer must check if the lamp has reached a stable level and if any fluctuations during the measurements are acceptable (stability 'within run'); furthermore the light energy may drift (stability 'within-days').

This control is possible by a measurement on air but should be done at different wavelengths, especially at 340 nm where the power of the tungsten lamp is low. Different instruments offer different advantages for this:

For the Dacos (Coultronics) and the Dimension (Dupont) the light source and photocells turn in front of the optical cuvettes.

For the Open (Biomerieux), the light source, with five optic fibres and filters and five independent photodetectors, are assembled on a rail resembling a printer head; these optics move in front of the cuvettes and measure 800 absorbances every 9 s at five different wavelengths for the 40 cuvettes.

(*a*) Within-run stability.

(*b*) Within-day stability.

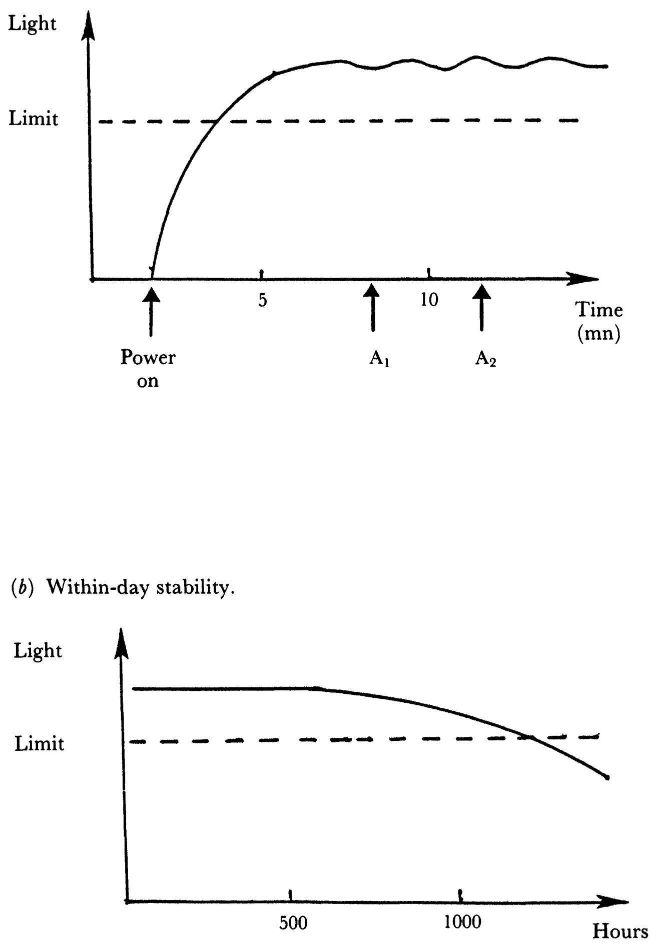

*Figure 1. Control of light sources – continuous mode.*

The *flash mode* is increasingly being used in analysers, with xenon flash lamps; their main advantages are a high light energy, a large spectral distribution from 300 to 800 nm, and a minimal effect on the thermal regulation systems of analysers. These lamps are also used for time-resolved fluorimetry techniques in immunoassays.

In these lamps, monitoring the pulse waveform is a problem as well as the duration of the flash, and the reliability of successive flashes.

### Control of monochromatic filters

The classic specifications of optic filters are absorbance wavelength, bandwidth and percentage of transmission. In random access analysers, the filters are often placed on a rotating filter wheel, and between the different measurements of one reaction, other measurements are made at other wavelengths. The position of the filter must be precisely controlled by a step-by-step motor to guarantee the homogeneity of the measurements.

### Control of optical cuvettes

*Glass cuvettes* are used in transfer systems like the Greiner G 400 and G 450, and in the American Monitor Parallel. The computer must check cleanness with a reagent blank, by measurement of empty cuvettes, or best of all by measurement on a cuvette filled with distilled water.

For *plastic cuvettes*, the most important potential interferences with the measurement of the reaction include: the absorbance of the material; the pathlength, which can be modified if the welding is not correct; the polished surfaces; and, for little cuvettes, the homogeneity of the optical cuvette and its position for repeated measurements of one reaction. See figure 2 for details.

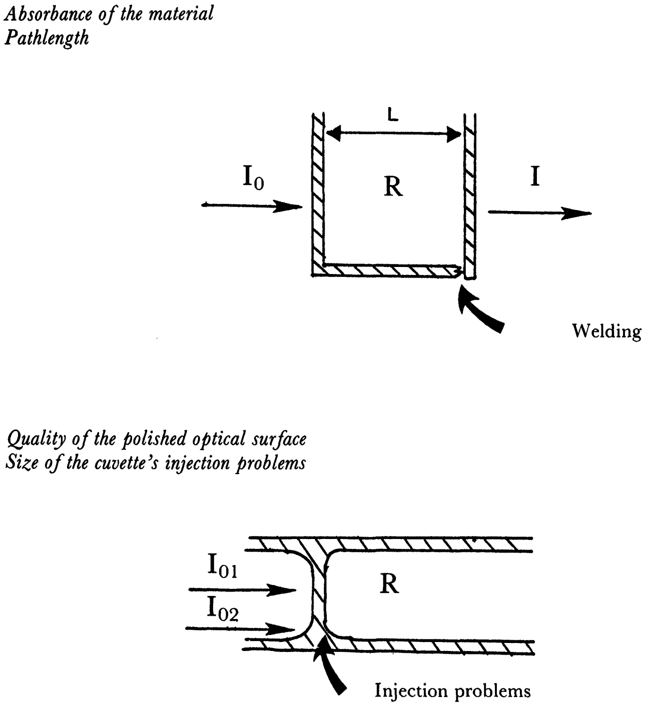

*Figure 2. Control of plastic cuvettes – injection moulded and ultrasonically welded.*

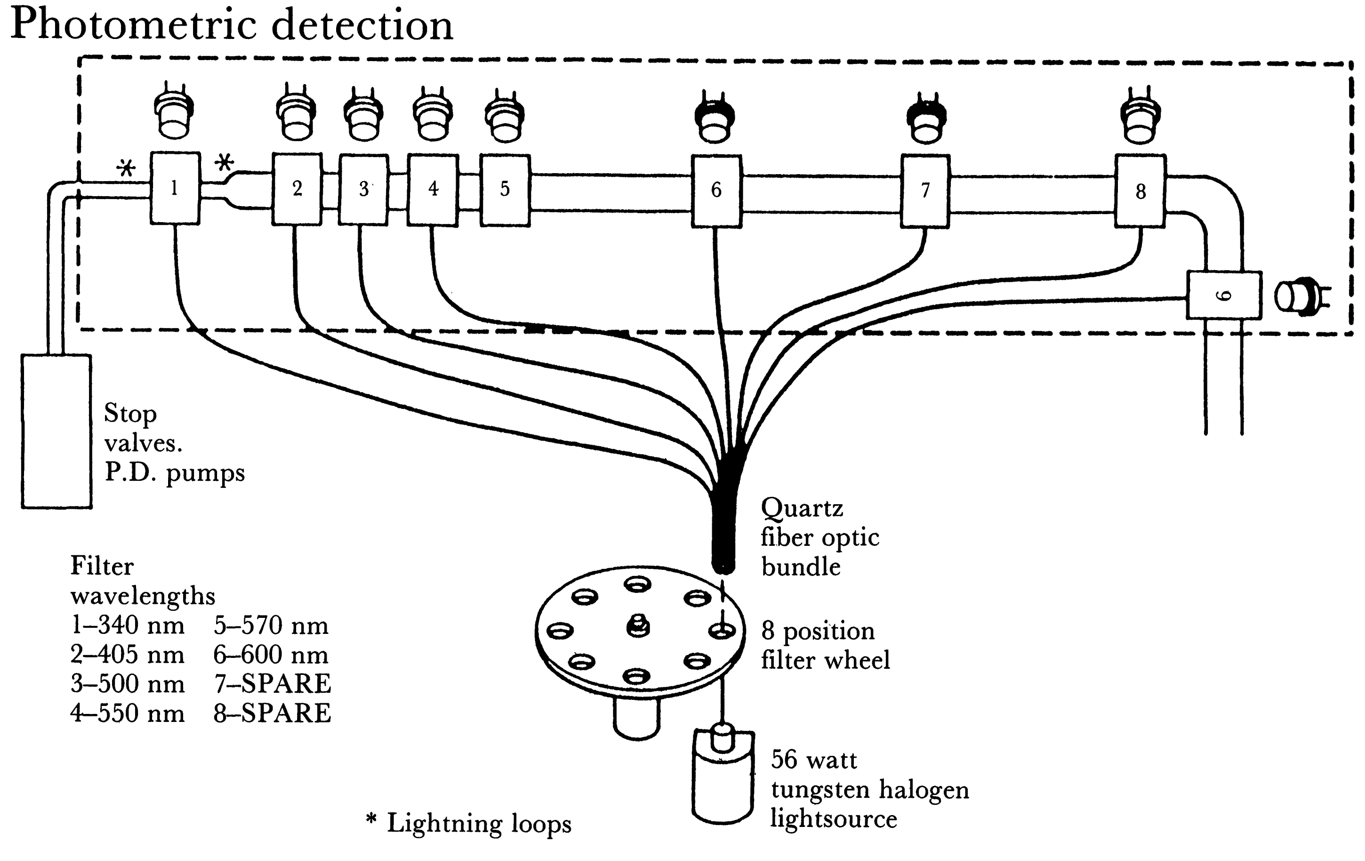

*Figure 3. Optics of the Chem 1 System.*

A way of avoiding these problems is to take measurements just after starting the reaction in centrifugal analysers and employing bichromatism in most analysers. Optical pathlength variations are avoided in several ways: for the Roche Cobas Fara, a centrifugal analyser with very sophisticated disposable cuvettes assembled to get a ring, the cuvettes lie longitudinally to the lightpath, avoiding the pathlength problem by variable pathlength measurements; on the Dupont Dimension system, the cuvettes are moulded *in situ* from a flexible film.

### Optical fibres

Optical fibres, or light-pipes, are used to guide light between the different components of the optics. This increases the flexibility of the instrument in that there is one light source for several cuvettes and the optical system can be placed in a location that affords easy access for maintenance and repair. In the Paramax (made by Baxter Travenol), which has moulded cuvettes in rolls and tabletted reagents, the optical system uses two filter wheels turning 1·800 r/mn to read absorbances coming by light pipes from eight photometric stations. In the Chem 1 (from Technicon) [1], which has capsule chemistry, different optical detectors are placed along a Teflon tube, the monochromatic light coming from optical fibres, allowing repeated measurements of one reaction mixture; this is shown in figure 3.

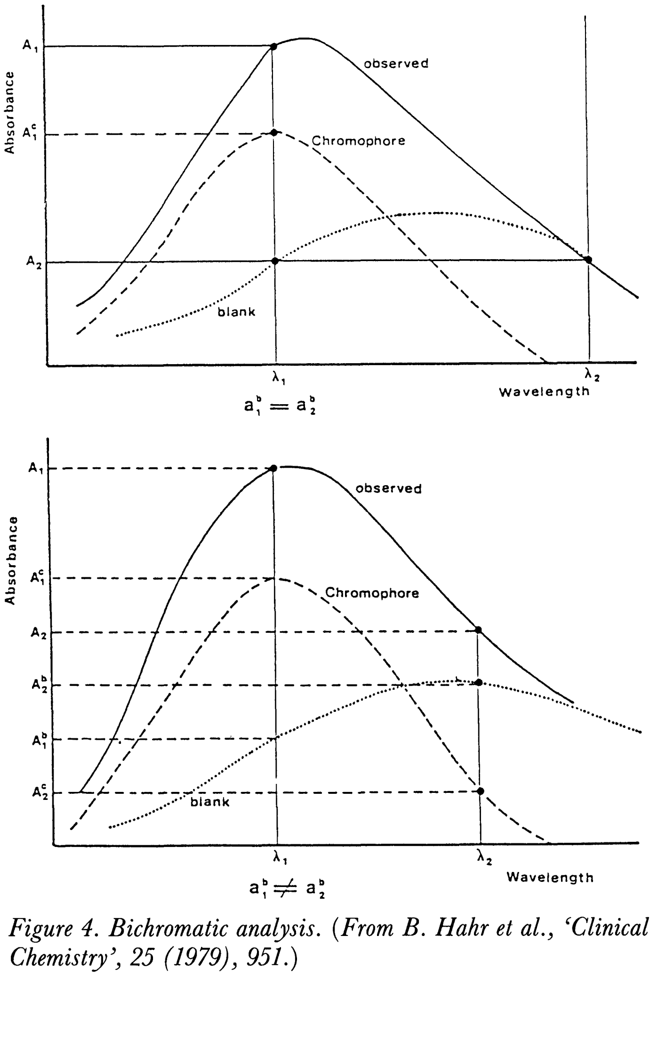

*Figure 4. Bichromatic analysis. (From B. Hahr et al., 'Clinical Chemistry', 25 (1979), 951.)*

### Polychromatic analysis [2]

Taking bichromatism first, figure 4 shows (top half) a 'perfect' case because the blank absorbs equally at λ 1, and λ 2, but generally case with serum samples, the bottom half of the graph applies $A^{b}_{1} \neq A^{b}_{2}$. So, bichromatism is useful only for checking electro-optical instability, variations in reagent concentration, and differences in cuvette absorbance. However, this can be the basis for polychromatic measurements to detect various interferences or to develop new analytical approaches [3].

In the Hitachi 737, automatic measurements of serum indexes were proposed to the user but the real polychromatic analyses are possible with linear diode array photodetectors, described in figure 5. Figure 5(*a*) shows a classic optic with a holographic grating – a motor selects the wavelength. Figure 5(*b*) is a diode array spectrophotometer, with the polychromatic light passing through the cuvettes and split by the grating. The complete spectrum is collected by a linear diode array photodetector (LDAP).

This optical system has no moving parts and can be completely closed. In the Abbott Spectrum and Vision analysers diode array spectrophotometers were developed. This optical system can measure the complete reaction profiling of a reaction against time. This technology allows also combination testing of two parameters.

*(a) Holographic grating.*

*(b) Diode array spectrophotometer.*

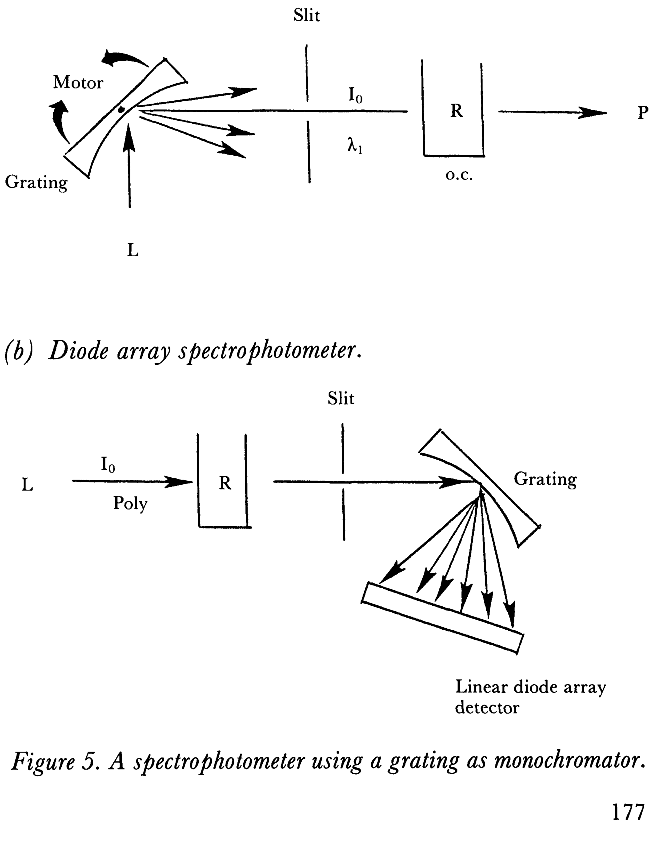

*Figure 5. A spectrophotometer using a grating as monochromator.*

A diode array spectrophotometer, allied to a powerful computer, allows calculation of first and second derivative spectra. Then, it is possible to eliminate background contributions for the chemical reaction, i.e. hyperlipaemic samples and coloured background in determinations on urine samples [3 and 4].

In conclusion, in random access analysers, optics are simpler, more compact, reliable and versatile. The microprocessor provides for fast data acquisition, management, reduction and display. These advances were often accomplished to limit errors due to the optics, mainly disposable cuvettes and also to increase the flexibility of measurement.

These advances open new analytical approaches, for instance simultaneous monitoring of multiple analytes, assessing possible interferents; performing sample and reagent blanks optically rather than chemically; and, finally, first- and second-derivative spectrometric techniques can be implemented.

### References

1. Cassaday, M., Diebler, H., Herron, R., Pelavin, M., Svenjak, D. and Vlastelica, D. *Clinical Chemistry*, **31** (1985), 1453.
2. Hahn, B., Vlastelica, D. L. Snyder, L. R., Furda, J. and Rad, K. J. M., *Clinical Chemistry*, **25** (1979), 951.
3. Burtis, C. A., *Clinical Chemistry*, **33** (1987), 352.
4. Levillain, P., *Informations Scientifiques du Biologiste*, **12** (1986), 131.
5. Merrick, M. F. and Pardue, H. L., *Clinical Chemistry*, **32** (1986), 598.

**THE STATE OF THE UK HPLC MARKET 1988–1990**

A comprehensive market survey of the UK HPLC market is now available from Finlay Robertson. The report contains 46 pages of detailed information about who leads the market-place in HPLC instruments and HPLC consumables and accessories.

There are 19 tables giving the names of all manufacturers and suppliers of HPLC equipment to the UK market. Market shares and market sizes are detailed.

*The price of the report is £395 or $695.*

*Full details of the HPLC Market Survey from Finlay Robertson, 5th Floor, Brook House, 77 Fountain Street, Manchester M2 2EE, UK.*

